# Intraspecific Crossability in *Andrographis paniculata* Nees: A Barrier against Breeding of the Species

**DOI:** 10.1100/2012/297545

**Published:** 2012-06-04

**Authors:** Alireza Valdiani, Mihdzar Abdul Kadir, Mohd Said Saad, Daryush Talei, Vahid Omidvar, Chia Sok Hua

**Affiliations:** ^1^Department of Agriculture Technology, Faculty of Agriculture, Universiti Putra Malaysia, Selangor, 43400 Serdang, Malaysia; ^2^Sime Darby Company, Centre Sdn. Bhd., Bukit Tangga, Kedah, 06050 Bukit Kayu Hitam, Malaysia; ^3^Department of Cell and Molecular Biology, Faculty of Biotechnology and Biomolecular Sciences, Universiti Putra Malaysia, Selangor, 43400 Serdang, Malaysia; ^4^Department of Crop Science, Faculty of Agriculture, Universiti Putra Malaysia, Selangor, 43400 Serdang, Malaysia

## Abstract

The ambiguity of crossability in *Andrographis paniculata* (AP) was pointed out in the present research. Accordingly, the effects of different style length and crossing time on intraspecific crossability of seven AP accessions in 21 possible combinations were investigated. The best results came out between 08:00 to 11:00 h for manual out-crossing of AP, while the time from 12:00 to 18:00 h showed a decreasing trend. Moreover, 12 mm style length was found as the most proper phenological stage in terms of stigmatic receptivity to perform out-crossing in this plant. All in all, AP behaved unlikely in each combination, and a significant difference was observed in crossability of AP accessions (*P* < 0.01). The lowest and highest crossability rate was found in hybrids 21 (11261NS × 11344K) and 27 (11322PA × 11350T) with 0.25% and 13.33%, respectively. Furthermore, a significant negative relationship between style length and crossibility (*r* ^2^ = 0.762^∗∗^) was recorded in this research. As a final conclusion, crossing time and proper style length can improve the intraspecific crossability in the species, considerably. Despite all the mentioned contrivances, we still believe that a genetic incongruity should be involved as an additional obstacle in crossability of those combinations that failed or responded deficiently to outcrossing.

## 1. Introduction

**Figure 1:**
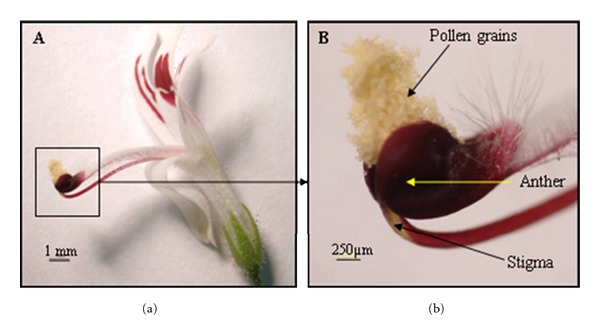
(a) An open flower of AP. Bar = 1 mm. (b) Close proximity of anthers and stigma where the stigma is inserted between the two-celled anthers. Bar = 250 *μ*m.

The plant species *Andrographis paniculata* Nees. (AP) belonging to the family Acanthaceae is an economically concerned important medicinal plant with a broad range of curative and pharmaceutical properties [1]. Synchronization of anther dehiscence and stigma receptivity in AP along with the flower structure in which intimate proximity of stigma with the two-celled anthers (Figures 1(a) and 1(b)) supports the plant to be a hermaphroditic, self-pollinated, self-compatible, and habitual inbreeder [2]. Additionally, the mating system of AP is categorized within the protandrous [3] and preanthesis cleistogamous plants [4]. Hypothetically, having a self-pollinating nature is the main reason to minimize the genetic divergence of the species [5]. Besides, the phenomenon autogamy (self-fertilization) is an ordinary mating system in plants and is known to decrease genetic diversity, increase genetic structure, and potentially put populations at greater risk of extinction [6]. So, the genetic composition of the plant in Malaysia has been tending to a low diversity situation. Intraspecific hybridization can be utilized as an alternative strategy to change the current trend [7]. Concurrently, the assay will allow a better understanding of the modality of heterosis, heritability, and combining ability of the important traits in the species. Moreover, many of the biometrical methods in the quantitative genetic analysis such as diallel are based on hybridization. Strategically, inter- and intraspecific hybridizations are considered as a beginning to produce new plant varieties. Unfortunately, none of these have been achieved yet, while the lower cross-compatibility (crossability) of AP accessions (as the main problem) has not been solved. However, there is no confirmed statement about the crossability of AP, but the low crossability rate of this species has been reported once, in which 32 out of 34 emasculated flowers on outcrossing, produced no seed [3]. Nevertheless, no further details have been declared in this regard. Unfortunately, intra-specific crossability is still an unresolved subject, because in general, AP accessions respond poorly when they are outcrossed with each other. The avoidance of accepting pollens of foreign individuals, cause a significant failure in intraspecific crossability. Therefore, crossability as a barrier becomes a research priority that must be bypassed in this approach. 

The mentioned problem led us to investigate the reason of this deficiency and to find a way to solve the case. The body of the methodology and underlying factors such as stigma receptivity, pollen viability, effect of out-crossing time, as well as the impact of accessions in different combinations (crosses), is built based on the similar disciplines applied to the other plants. The collection and analysis of data have been processed in light of other research findings. The present study is the first-ever report on deliberate intraspecific hybridization between *Andrographis paniculata* accessions in a large scale (over 8000 of crosses) that emphasizes the phenological aspects of this problem. From another point of view, this is the first step towards the classical breeding procedure of AP.

## 2. Material and Methods

### 2.1. Plant Materials

A total of 400 plants consisting of seven AP accessions representing six states of Peninsular Malaysia were grown to be used as the parental individuals for one-way manual crossing (♀ × ♂). Since, the number of combinations using accession 11179 S (as mother plant) was higher than others, and that is why a sum of 100 individuals was developed for this accession. The geographical distribution and local name of these accessions are displayed in Table 1.

### 2.2. Growth Condition and Field Trial

High contamination and low germination are two prevalent problems in AP. To overcome these problems, the seeds of seven parental accessions were sterilized by soaking in 10% sodium hypochlorite (NaOCl) solution for 10 min in separate Petri dishes with 15 cm diameter. Whatman no.2 filter papers were used as the seedbed for this stage. The Petri dishes were sealed with parafilm to prevent any water loss and infection during incubation. The average temperature within the growth chamber was set between 28°C and 30°C, and the relative humidity (RH) varied between 60% and 75% [8]. Ten-day seedlings were transferred into the jiffy medium at the two leaf stage. The second transformation was conducted after thirty days when the young seedlings were at 6–8 leaf stage. To ease the plants' relocation, the seedlings were transferred into 22 × 35 cm polybag and to reduce the soil environment's effects on the crossing performance, contained of the polybags that were homogenized with fine sand, topsoil, and organic material at the ratio of 2 : 1 : 1. Macro- and microelement-enriched WELGRO compound fertilizer (NPK 15 : 30 : 15) was applied four times during the growth period. 

### 2.3. Pollen Viability Test

Pollen viability was tested using IKI (iodine + potassium iodide) which is more sensitive and faster in comparison with acetocarmine stain [9]. A completely randomized design experiment (CRD) was employed with four treatments and four replicates. The treatments were pollens from different bud stages, including (a) unopened anthers (9 mm bud), (b) fresh and mature pollens of unopened anthers (11 mm unopened bud), (c) pollens shortly after anther dehiscence (13 mm unopened bud), and (d) non-fresh pollens (15 mm opened bud). The samples were collected in Petri dishes separately, and pollen grains were placed on a glass slide by squeezing the anthers using forceps. Forceps was dipped in 70% ethanol in the interval in order to prevent pollen contamination of different samples. The grains of pollen were counted to determine viability after a couple of minutes in the IKI medium (1 g KI and 0.5 g I dissolved in 100 mL-distilled water) [9]. Cover slip was then gently placed over the stained pollens to avoid production of air bubbles. Transparent nail polish was applied on the edges to seal the cover slip and prevent desiccation. The slide was visualized under a light microscope with 10x magnification and was photographed using a built in-digital camera.

### 2.4. Stigma Receptivity Test

Hydrogen peroxide (peroxide water) assay was used for stigma receptivity test. In tests based on staining the stigma with hydrogen peroxide (peroxide water), it is assumed that the rate of bubble production from the stigmatic surface in hydrogen peroxide is directly related to the level of stigma receptivity [10]. Therefore, if the peroxidase enzyme is present, many oxygen bubbles will be released by the chemical reaction of the peroxide with the enzyme [11]. A preliminary design was used to evaluate the level of oxygen generation. The grading scale of 1–5 was developed to rank receptivity, so that 1 = no bubble production while 5 = very rapid bubble production [10]. The test was carried out on a factorial randomized block design with two factors and five replicates. The factors were seven different accessions (seven levels) and style length with six levels (9, 10, 11, 12, 13, and 14 mm). In order to ensure the freshness of stigma, the test was conducted under lab condition. The styles were immediately rinsed with distilled Millipore (high-performance liquid chromatography) HPLC water after emasculation to wash away pollen grains on the stigmatic surface. Stigma receptivity was determined based on stigmatic peroxidase activity (SPA) using 3% hydrogen peroxide [10]. The style was placed on a glass slide. A drop of 3% hydrogen peroxide was applied to the stigma and style as well using a micropipette. Cover slip was then gently placed over the sample to avoid air bubbles that could confound with the treatment effect. Bubbles production from the stigma within a minute was monitored under the binocular microscope. Bubbles production on the stigmatic surface is due to the presence of enzyme peroxidase [12]. 

### 2.5. Emasculation and Hand Pollination

**Figure 2:**
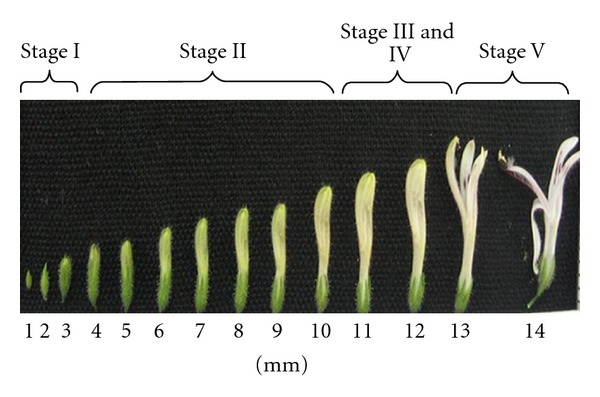
The sequential floral development of AP associated with floral stages, illustrating the development of newly emerged flower bud (1 mm) to fully open flower (14 mm).

**Figure 3:**
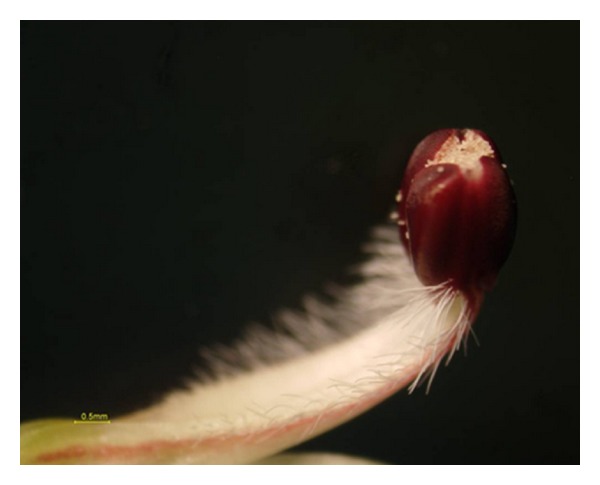
The two-celled anthers of AP at the length of 11.5 mm start splitting open, disclosing minute light yellowish pollen grains. Bar = 0.5 mm.

**Figure 4:**
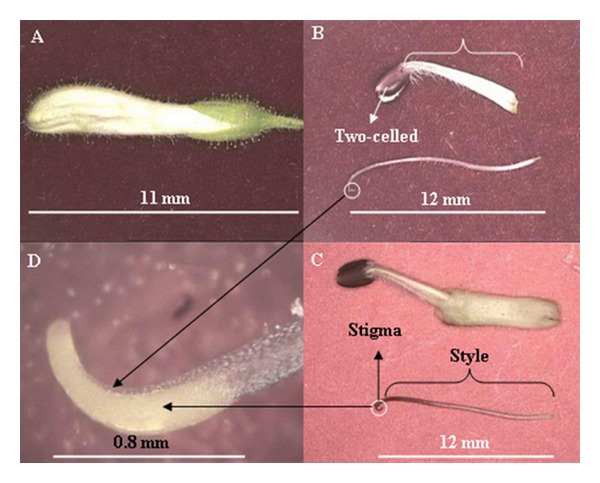
(A) The 11 mm unopened flower bud of AP, (B) an incompletely emasculated anther and separated style (if a part of petal or the filament will be left along with the style after emasculation, this will make the stigma to fall due to the imposed weight by the dried filament and petal, (C) a complete emasculation while the 12 mm emasculated anther and petal are entirely removed, and (D) the magnified image of the sticky stigmatic surface of a 12 mm style.

Previously, AP's floral development and maturity of anthers and stigma were studied by Chia [4], and the process was categorized into five stages based upon flower bud length (Figure 2). With peers on these results, anther dehiscence starts when the flower bud is 11.5 mm and thereafter (Figure 3). Hence, all flower buds were emasculated carefully by using sterilized forceps before anther dehiscence at 11 mm (the style length was almost 12 mm in this stage as the style is curved at the stigma part), so that the two-celled anthers were removed to prevent accidental self-pollination (Figure 4). After emasculation, the stigmas were precisely checked using handglass, so that the contaminated stigmas with pollen were eliminated from the hand-crossing process while the clean stigmas of the 12 mm styles were immediately handedcrossed by fresh pollens, and the whole plant was covered by micro-mesh to avoid unwanted pollen contamination. Besides, in rare cases that the style length was less than 12 mm, after emasculation, we covered them with micromesh and gave them the opportunity to reach to the ideal length. As it was mentioned, the stigmas of 12 mm styles were immediately handedcrossed by fresh pollens, and the whole plant was covered with suitable size of micronets, consequently. To protect the healthy 12 mm emasculated styles, the whole plant was covered by micro-mesh until they reached soon (a few hours after emasculation) to 13 mm and 14 mm of length and were hand-pollinated properly at different periods of time as the second and third levels of the style length. The netting was repeated after handpollination to protect the pollinated styles from any secondary contamination. In order to ensure the freshness of stigma, the handpollination was conducted under shade condition. The same procedure was repeated in all 21 possible combinations among the seven parental individuals. The numbers from 8 to 28 were given as identification codes to the hybrid progenies from 1 × 2 to 6 × 7, respectively (Table 3). The high failure rate of manual emasculation, especially in the first days and exceedingly weak response of AP to deliberate out-crossing, led us to operate a huge number of emasculations (over 10000) and 8077 of handcrosses. This process was continued for four months from June to September 2007 nonstop.

### 2.6. Main Experimental Design

After conducting the above-mentioned preliminary experiments, to achieve the main object of this study, which was determining the effect of cross-combination (accession), floral morphology (style length) and crossing time on intraspecific crossability of AP, a factorial design based on randomized complete block design (RCBD) with three factors including cross-combination (21 levels), style length (3 levels), and crossing time (12 levels) with 10 replicates was used for this study. Fruit set was used to determine the degree of crossability among AP accessions.

### 2.7. Levels of Used Style Length

Since the use of style length instead of flower bud length was friendlier, to simplify the hand-pollination, we used the style length as an applicable index to express the stigma receptiveness of AP, in this study. Subsequently, based on the result of stigma receptivity test (will be presented in Section 3), a clear and distinct pattern of stigma responses was obtained from the hydrogen peroxide test. Hence, to comply with this, the most receptive stigmas including three levels of style length (12, 13, and 14 mm) were subjected to handpollination in this exploration. Even though, all emasculations were carried out at 11 mm bud length, the handpollination was performed appropriately at the three mentioned style length's level (including 12, 13, and 14 mm) with transferring fresh pollens onto the stigmas followed by tagging and netting.

### 2.8. Handpollination Timing (Levels of Out Crossing Time)

Manual hybridizations were performed at 12 levels of time. To facilitate such a condition, firstly, 24 hours of a day were divided into 12 of two-hour periodic groups from 00 : 00–02 : 00 h to 22 : 00–24 : 00 h. A sum of 10 emasculated flower buds (as 10 replicates) was hand-pollinated at every of the mentioned 12 timings (120 crosses) for each style length separately (12 mm, 13 mm and 14 mm). As a result, a total of 360 (120 × 3) handcrossings were organized for each combination. Due to the complete failure of hand-pollinating (no fruit set) of two combinations (2 × 6 and 3 × 6) at the first place, a total of 242 and 275 extra hand-crossings in 12 mm style length were carried out on the above-mentioned crosses, respectively (Table 3).

### 2.9. Statistical Analysis

Data of pollen viability and stigma receptivity were subjected to analysis of variance using SAS software version 9.1 (SAS Institute Inc 2003). Duncan's multiple range test was performed for means comparison at *α* = 0.05 and 0.01. Due to the nonparametric nature of the crossability study, the Kruskal-Wallis test [13] was employed in this part of the research. SPSS software version 19 was utilized to run the latter test.

## 3. Results

### 3.1. Stigma Receptivity and Pollen Viability in AP

The result of stigma receptivity test, due to hydrogen peroxide activity and bubble releasing intensity, highlighted a clear border between weak and strong stigma receptivity in AP. However, the weak reaction was obtained from 9, 10, and 11 mm style lengths while the strongly positive responses rose from 12 mm, 13 mm, and 14 mm style lengths. However, stigma receptivity showed a nonsignificant difference among the accessions, but a significant difference at 1% level was recorded for stigma receptivity in the studied style lengths (Table 2). This situation, suggesting a full receptiveness of stigma commenced at 12 mm style length when the surface of stigma was at 12 mm of style length (shown in Figure 4(D)), where the stigmatic surface physically seems sticky as well. These outcomes can be considered as the best morphological (or even phenological) index toward the choice of the best stage that the female organ of AP's flowers is ready to be outcrossed artificially. This is what exactly led us to choose the 12, 13, and 14 mm styles as three levels of efficient style lengths in this study.

**Figure 5:**
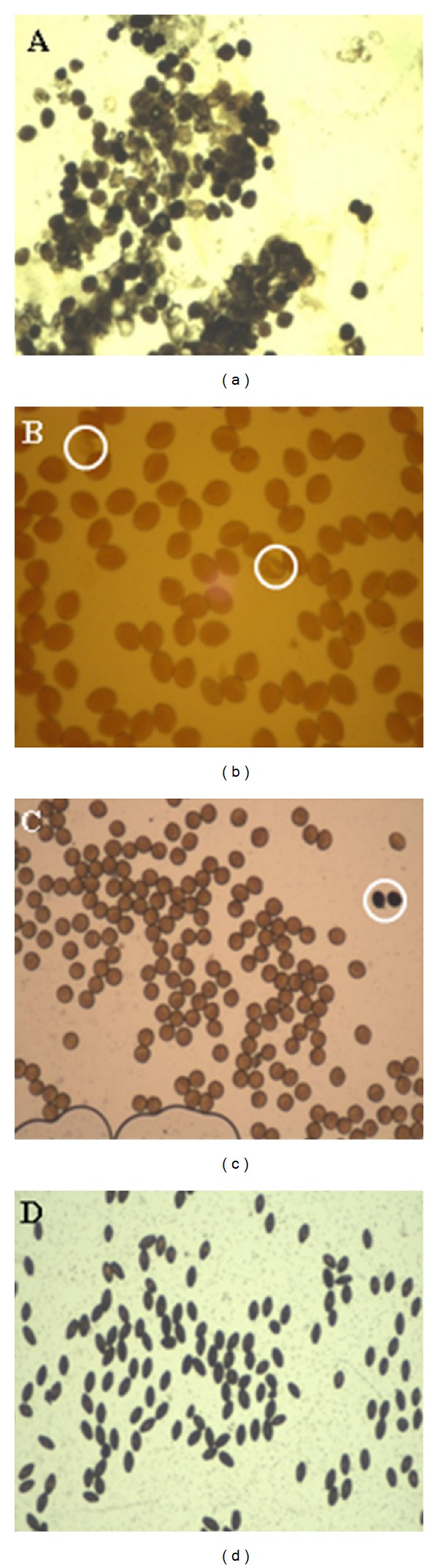
Pollen viability test of AP pollen grains sampled in different stages using IKI stain. Image (a) unstained pollens are immature (non-viable) pollens of unopened anthers (bud length 9 mm) while the black colour is due to the presence of starch in pollens, (b) dark-brown colour indicates the fresh and mature (viable) pollens of unopened anthers (bud length 11 mm), (c) viable pollens shortly after anther dehiscence (bud length 13 mm), and (d) shrunken and stale (non-viable) pollens (15 mm opened flower buds exposed to sunlight and rain drops). Pollen grains inside the yellow circles in images (b) and (c) are referred as non-viable pollens.

Unstained or shrunken pollen grains were recorded as nonviable, and red-stained pollen grains were considered viable. Black color, unstained and shrunken pollen grains refer all as non-viable pollens while brown-pollen grains are considered viable (Figure 5).

Eventually, it was confirmed that the unopened flower buds with length above 10 mm can be used as a safe source of viable pollens in any hybridization program of AP. Nevertheless, pollens that probably have been exposed to sunlight or rain for some time should be avoided.

### 3.2. Effect of Style Length on Crossability of AP Accessions

**Figure 6:**
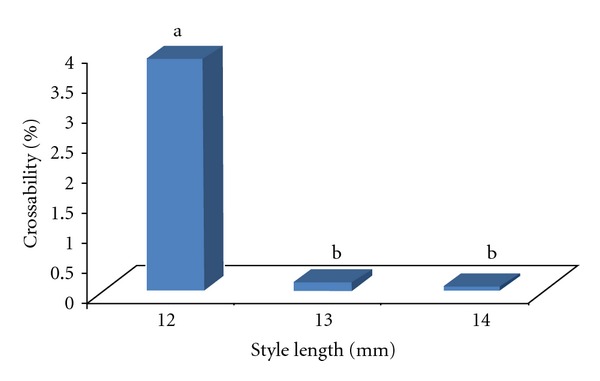
Crossability in different style length (%).

The results of this part of study complied with great impact of style length and stigma receptivity but in a different direction. As a preliminary experiment (other than the main experiment), consisting of 900 crosses between accession 11179 and 11216 of which were equally distributed (300 crosses on each style length) on the stigmas with 9, 10, and 11 mm style produced only one fruit (capsule) at style length 11 mm (Results non shown). The weak response of AP to 9–11 mm style lengths was completely predictable as it was in agreement with the stigma receptivity test's results. However, the unexpected results came out from the main levels of style length while, except the 12 mm styles, conducted crosses on the 13 and 14 mm styles almost failed (Figure 6). Hence, this part of the results disagreed with the stigma receptivity test which means that, unlike our initial prediction, the completely receptive stigmas with 13 and 14 mm length refused to accept the viable pollen grains during the handpollination process. From a statistical point of view, the Kruskal-Wallis test revealed that the effect of style length, which is used as an index to express the stigma receptivity in this study, poses an important role in the crossability of AP. This effectiveness is reflected in the significant chi-square value (186.267**) shown in Table 4.

### 3.3. Effect of Handpollination Time on Crossability of AP Accessions

**Figure 7:**
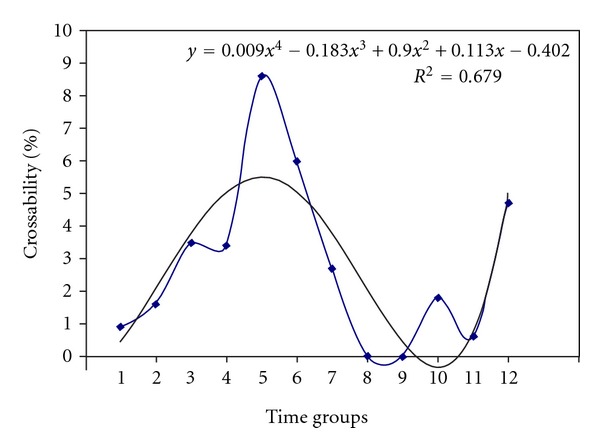
The trend of crossability during 24 hours of a day.

The outcomes of this study proved the undeniable importance of hand-crossing time (cross timing) and its effect on increasing the chance of crossability. As a matter of fact, AP's feedback to conducting intraspecific manual-hybridization was positively increased from late evening time until early morning. This trend was inversely changed from noon time until evening. According to the results, the time between 08 : 00 and 11 : 00 h was identified as the best time for manual crossing in AP while 12 : 00 to 18 : 00 h showed a decreasing trend of crossability so that cannot be considered suitable to perform hand-crossing (Figure 7). No wonder that the effect of time on crossability of AP accessions complied with the result of Kruskal-Wallis test when a significant chi-square value (234.326**) came out accordingly (Table 4).

### 3.4. Crossability of the Accessions in Different Combinations

**Figure 8:**
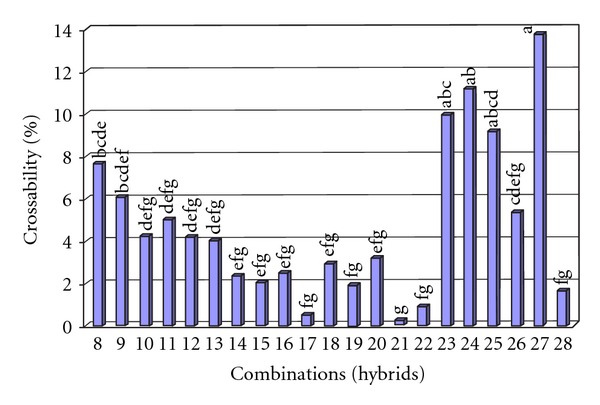
The percentage of crossability in 21 combinations.

Finally, we realized that the crossability of AP accessions can be affected by factors such as crossing time and stigma receptivity though, but, despite being provided by the best conditions, still some accessions barely responded to manual crossing. Anyhow, crossability among AP accessions in intraspecific manual hybridization was ranged with a significant difference from 0.25% to 13.33% in combination 21 (hybrid 3 × 6) and combination 27 (hybrid 5 × 7), respectively (Figure 8). The percentage of abortions in each combination has been presented in Table 3. The Kruskal-Wallis test approved the significance of the accession's effect or so-called combination's effect (84.573**) on the crossability of AP (Table 4).

## 4. Discussion

Style-stigma complex and style lengths both have been used for the studies related to pollination biology and seed set of self- and cross-pollinated plants. Some literatures have postulated that figs control seed loss though variation in style length [14–18]. In some plants such as fig (*Ficus maxima*), it is believed that stigma length shows an allometric relationship with style length. This is probably explained by their larger stigmatic surface that increases their chance of receiving the passively dispersed pollen [19]. However, at least about AP, this aspect of the style/stigma size relationship was not concerned. As it was mentioned before, we tried to use the style length as an applicable floral-morphological index to introduce the best time of manual crossing.

In spite that, stigma receptivity and pollen viability of AP both have been investigated by Chia [4]; hitherto, the impact of stigma receptiveness (expressed by style length) on intraspecific crossability of the species has never been explored. However, Chia [4] had used acetocarmine for pollen staining, but the result of this study was in agreement with her upshots as no significance difference was observed in pollen viability of pollens supplied from the seven parental accessions (*P* > 0.05), while the viability of pollens sampled in different stages varied significantly (Table 2). In addition, the pollen viability test using a new approach (IKI), not only proved the previous results, but it also disclosed some new points. A dark bluish-black color (stained with IKI) indicates that the presence of starch in pollens but the birefringence of starch, which is due to the crystalline properties of amylose, is not a constant characteristic [20].

According to Chia [4], AP's stigma showed a positive but weak response from 8 mm to 11 mm flower buds while the stigmas behaved fully receptive once the flower bud reached to 12 mm. The weak responses indicated that stigma was partially receptive. The result of the current study was in a complete agreement with the previous experience, yet the unexpected part of the results was recorded when crossability failed in fully receptive stigma levels. A reasonable explanation in this regard is that the stigmatic surface became dried after emasculation because, after removing the petals, they were exposed to the air. As a matter of fact, the time gap between emasculation and hand-crossing of the stigmas with 13 and 14 mm styles gives rise to the conflict between the result of stigma receptivity test and real crossability performance of the fully receptive stigmas. In other words, in stigma receptivity test, the fresh stigmas (with 12, 13, and 14 mm styles) were immediately subjected to hydrogen peroxide while, in practice and during the hybridization procedure, the stigmas of 13 and 14 mm styles were cross-pollinated about 6 to 12 hours after emasculation, respectively. The shock that pistil might receive during emasculation procedure could be debated as another reason behind this approach.

Practically, a yellowish-powdery source of pollen provided from unopened flower buds (11–13 mm) can satisfy any breeder to conduct any hybridization-based project on AP. Unlike pollen, the role of stigma can be challenged seriously.

The impact of light and temperature on crossability (seed set) should be concerned as the main environmental factors which induce their effect in time of crossing as it has been studied on different plants [21]. However, the results are almost the same, for example, in rice increasing night and day temperature both reduce seed set [22], while availability of light has a positive effect on fruit set of *Styrax obassia* [23]. In mulberry, different colors of light affect the seed set and seedling behavior in different ways. Seed set of mulberry was higher in red and orange light and less in darkness [24]. Light level can directly and indirectly affect the plant reproductive success [25]. Perhaps the existence of the similar situation makes the feedback of AP to comply with a higher crossability rate among different accessions at early hours during the day when the light was available but the temperature was not that high.

Another interpretation for this can be justified by the presence of an elevated concentration of atmospheric carbon dioxide (CO_2_), light and cool temperature in the early morning which causes a direct increase in photosynthesis and an indirect increase in seed set [26]. Respiration process in plants during nighttime is the source of this accumulated CO_2_. In bryophytes, condensation resulting in dew provides moisture from the surface of plants and can rehydrate them from the desiccation of daytime. Such moistening will reach its maximum just before dawn, preparing the bryophytes to take advantage of the cool temperatures in the early-morning light. Csintalan et al. [27] demonstrated this phenomenon in the desert moss *Syntrichia ruralis*. Despite that AP is not a bryophyte, yet due to a general principle the plant can take an advantage of adsorbing the atmospheric H_2_O and CO_2_ into the photosynthesis cycle as the most necessary substrate upon to receive the first beams of light during sunrise while the temperature is still in an optimum level. Hence, the photosynthetic base of the plant will be strengthened by this. In addition, the optimum amount of relative humidity at the morning time will facilitate the stability of the transmitted pollen grains on the stigmatic surface by maintaining the stickiness of stigma. Although this will give an opportunity to the pollens where it hydrates, germinates, and extends a pollen tube [28], in contrast to an optimum level of atmospheric H_2_O, a saturated level of water in the air will act as an inhibitor factor to fruit set and consequently for crossability. The vapor pressure deficit phenomenon (VPD = difference between saturated and present humidity conditions) and high CO_2_ levels promote fruit set as a result of improved photosynthesis increases fruit set [29]. Further improvement of fruit set possibly might be obtained by maintaining a low humidity during the later part of the night and the early-morning hours to improve the release of the pollen from the anthers, followed by a high humidity during the latter part of the day [29]. Presumably, for the same reason, a high rate of crossability did not happen during nighttime as the humidity condition (saturated humidity) logically is more than daytime. To prevent fruit set, a low humidity by day could be combined with a high humidity by night [29].

On the other hand, our outcomes agreed with the result of other studies in which the rate of crossability was decreased by increasing the temperature at noon time and early afternoon. High temperatures (35°C<) completely hamper fruit set of pepino (*Solanum muricatum* Aiton) especially in nonparthenocarpic clones [30]. It is believed that high temperatures reduce the pollen fertility; however, in our case there is possibility that the stigmatic surface has been dried and became nonreceptive due to high temperatures.

A final conclusion can be constructed in which the style length (stigma receptivity) and out-crossing time can increase the chance of crossability, and this will be helpful in breeding more prolific varieties or at least to study the genetics of the herb. Early morning and exactly the time between 08:00 and 11:00 o'clock are the best time to conduct out-crossing on AP and any unopened 11–13 mm flower bud can be a safe source of viable pollen. 12 mm styles are the best option with the most receptive stigmas. Despite all these contrivances, the great influence of unknown genetic factors on the intraspecific crossability in this species is undeniable. Furthermore, we used 12 levels of crossing time in the present study, but, indeed, the factor of crossing time was used as a middle tool to extract the effect of important environmental factors such as light, temperature, RH and CO_2_ level on crossability without any specific measurements on these factors separately. In other word, out-crossing time was a shortcut to this approach. All in all, prescreening using molecular assays will reveal valuable information about genetic structure of AP population so that can be utilized in the next hybridization and breeding programs. However, our hypothesis is that crossability of AP is extremely sensitive about genetic incongruity even in slightest levels.

## Figures and Tables

**Table 1:** List of parental AP accessions from Peninsular Malaysia.

Code*	Accession number	State	Vernacular name	No. of plants	Latitude	Longitude	Altitude (M)
1	11179S	Selangor	Tutup Bumi	100	2° 56. 454 N	101° 26.020 E	−20
2	11216NS	Negeri Sembilan	Hempedu Bumi	80	5° 03.358 N	100° 30.672 E	−32
3	11261P	Perak	Akar Cerita	55	5° 04.610 N	100° 23.561 E	−39
4	11313PA	Pahang	Hempedu Ular	50	3° 00. 242 N	101° 46.952 E	−7
5	11322PA	Pahang	Hempedu Bumi	45	3° 37. 851 N	101° 02.759 E	−1
6	11344K	Kelantan	Lidah Ular	40	3° 37. 851 N	101° 02.759 E	−1
7	11350T	Trengganu	Lidah Ular	30	2° 55. 864 N	101° 27.415 E	−27

*Given code in this study

**Table 2:** Analysis of variance for stigma receptivity and pollen viability of seven AP accessions.

Source	df	Mean square	Source	df	Mean square
		Stigma receptivity			Pollen viability
Accession (A)	6	0.038^ns^	Accession (A)	6	4.195^ns^
R	4	0.088^ns^	R	3	13.502^ns^
Style length (SL)	5	107.973**	Bud length (BL)	3	83126.172**
A × SL	30	0.027^ns^	A × BL	18	6.341^ns^
Error	164	0.093	Error	111	5.443

**Significant in 1% level, ns: non-significant.

**Table 3:** Hybridization scheme between AP accessions.

Code	Hybrid	Pistillate ♀	Staminate ♂	Number of crosses	Abortion (%)
8	1 × 2	11179S	11216NS	360	92.50
9	1 × 3	11179S	11261P	360	94.17
10	1 × 4	11179S	11313PA	360	95.83
11	1 × 5	11179S	11322PA	360	95.00
12	1 × 6	11179S	11344K	360	95.83
13	1 × 7	11179S	11350T	360	95.83
14	2 × 3	11216NS	11261P	360	98.33
15	2 × 4	11216NS	11313PA	360	98.33
16	2 × 5	11216NS	11322PA	360	97.50
17	2 × 6	11216NS	11344 K	602	99.45
18	2 × 7	11216NS	11350T	360	97.50
19	3 × 4	11261P	11313PA	360	98.33
20	3 × 5	11261P	11322PA	360	96.67
21	3 × 6	11261P	11344K	635	99.75
22	3 × 7	11261P	11350T	360	99.17
23	4 × 5	11313PA	11322PA	360	90.00
24	4 × 6	11313PA	11344 K	360	89.17
25	4 × 7	11313PA	11350T	360	90.83
26	5 × 6	11322PA	11344K	360	95.00
27	5 × 7	11322PA	11350T	360	86.67
28	6 × 7	11344K	11350T	360	98.33
Total	21	—	—	8077	—

**Table 4:** Nonparametric analysis using Kruskal-Wallis test.

	Combination	Time	Style length
Chi-square	84.573**	234.326**	186.267**
df	20	11	2
Mean ± Std.	18.07 ± 5.89	6.44 ± 3.35	12.94 ± 0.83
Sig.	0	0	0

**Significant in 1% level.
